# Does CD47 expression have prognostic significance in classical Hodgkin lymphoma?

**DOI:** 10.55730/1300-0144.5958

**Published:** 2024-12-30

**Authors:** Aydan KILIÇARSLAN, Gülnaz KURT ÇEVİK, Mehmet DOĞAN, Ayşegül AKSOY ALTINBOĞA, Funda CERAN, Gülşah EFECİK, Mine BAKANAY ÖZTÜRK

**Affiliations:** 1 Department of Pathology, Faculty of Medicine, Ankara Yıldırım Beyazıt University, Ankara, Turkiye; 2 Department of Pathology, Ankara City Hospital, Ankara, Turkiye; 3 Department of Hematology, Ankara City Hospital, Ankara, Turkiye; 4 Department of Hematology, Faculty of Medicine, Ankara Yıldırım Beyazıt University, Ankara, Turkiye

**Keywords:** Classic Hodgkin lymphoma, CD47, Epstein-Barr virus

## Abstract

**Background/aim:**

Hodgkin lymphoma (HL) is highly treatable, but new chemotherapy agents are needed for patients with progression or recurrence. CD47 regulates antiphagocytic activity by signaling through the signal regulatory protein alpha (SIRPα) pathway on macrophages. This study aimed to assess the expression of CD47 in HL cases and explore its relationship with Epstein–Barr virus (EBV) status and the International Prognostic Index (IPI).

**Materials and methods:**

One hundred twenty patients diagnosed with classical HL, characterized by the presence of Hodgkin and Reed–Sternberg (HRS) cells, and for whom IPI scores were available were included. Their demographic data and EBV status were retrieved from hospital records. CD47 immunohistochemical expression in HRS cells was evaluated based on its prevalence and intensity. The product of these two values was used to calculate the CD47 expression score.

**Results:**

Of the 120 patients, 76 (63.33%) were male and 44 (36.66%) were female, with an average age of 38.77 years. EBV ISH test results were available for 93 patients, of which 48 (51.61%) were positive. CD47 expression was 67% on average, and patients were categorized based on CD47 expression scores of ≤6 and >6 and IPI scores of ≤2 and >2. No significant relationship was found between CD47 expression score and age (p: 0.990), sex (p: 0.086), EBV status (p: 0.374), or IPI score (p: 0.805). However, higher IPI scores were significantly associated with increased EBV positivity (p: 0.041).

**Conclusion:**

CD47 was widely expressed, but no significant relationship with clinical parameters was observed. Further studies are required to validate its role as a biomarker.

## 1. Introduction

Hodgkin lymphoma (HL) is a B-cell lymphoma that constitutes 15% of all lymphomas. Although it is largely treatable, achieving adequate treatment outcomes in advanced-stage cases remains a challenge [1,2]. Additionally, there is no consensus on the treatment plan for early-stage patients, and the prognosis of patients showing recurrence is unfavorable. New agents are being researched to reduce the number of chemotherapy agents and their toxicity [3]. CD47 is a receptor with immunoregulatory functions, expressed on the surface of the cell membrane in numerous cell types. Through its ligands (thrombospondin-1 (TSP-1), signal regulatory protein alpha (SIRPα), integrin, and SH2-domain bearing protein tyrosine phosphatase substrate-1 (SHPS-1)), CD47 mediates cellular phagocytosis by macrophages, neutrophil migration, dendritic cell immune activation, and interactions between T and B lymphocytes [4]. Activation of the CD47–SIRPα axis allows tumor cells to evade phagocytosis and escape immune surveillance, thereby promoting tumor progression. The expression of CD47 on the surface of neutrophils, dendritic cells, and T cells triggers reverse signaling pathways, neutralizing immune responses against tumors. CD47 with TSP-1 shows direct anticancer and antiangiogenic effects [5]. Studies in solid tumors and various hematopoietic malignancies (such as leukemia, lymphoma, and multiple myeloma) have detected CD47 expression and found it associated with poor clinical outcomes, regardless of expression level [6–10]. Epstein–Barr virus (EBV) is observed in approximately 40%–50% of classic HL cases [11]. However, the contribution of the EBV to the neoplastic process in tumor cells has not been clearly explained [12]. In our study, we aimed to determine the expression status of CD47 in HL patients and investigate its relationship with EBV status and the International Prognostic Index (IPI).

## 2. Materials and methods

### 2.1. Study population

A total of 321 patients diagnosed with classic HL between 2010 and 2022 were identified. Of these, 136 had been diagnosed through excisional biopsy. Excisional biopsy preparations were retrieved from the archives and reexamined according to the 2022 World Health Organization classification. Patient sex and EBV encoded RNA (EBER) test results were obtained from the hospital information management system. Additionally, data such as age, Ann Arbor Stage, ECOG performance status, serum LDH levels, and extranodal site involvement were accessed, and the IPI score was calculated. Ten patients for whom the IPI score could not be determined and six lacking sufficient lymphoma tissue were excluded, leaving 120 in the study. The study received approval from the Ankara Bilkent City Hospital Ethics Committee on 15/11/2023 with protocol number E1-23-4302 and was conducted in accordance with the Declaration of Helsinki.

### 2.2. Preparation of tissue microarrays (TMAs)

The areas most representative of HL and containing the most Hodgkin and Reed–Sternberg (HRS) cells were marked on the preparations. From these areas, TMA blocks were prepared using 4-mm-diameter tissue cores.

### 2.3. Immunohistochemical and ISH Staining

Sections 3 μm thick were cut from the TMA paraffin blocks obtained from lymphoid tissue on a microtome and placed in an instrument for immunohistochemical staining. Deparaffinization and incubation were performed using a BOND-MAX Fully Automated IHC and ISH Staining System. All cases were stained using the CD47 immunohistochemistry marker (mouse monoclonal B6H12, catalog no: 14-0479-82, Invitrogen Thermo Fisher Scientific, 1:100 dilution). The tissue used as a positive control for CD47 immunohistochemical staining is placenta (trophoblastic and endothelial cells). CD47 immunohistochemical staining is evaluated exclusively on Hodgkin and HRS cells, without consideration of surrounding lymphoid background cells. The percentage of stained HRS cells was scored between 0 and 4 (0: <1%, 1: 1%–10%, 2: 11%–33%, 3: 34%–66%, 4: ≥67%) and staining intensity was scored between 0 and 3 (0: none, 1: weak, 2: intermediate, 3: strong). According to the immunoreactive score (IRS) system, the multiplication of these two values was accepted as the CD47 expression score (range 0–12). EBER in situ hybridization was performed on the same instrument using fluorescent-labeled oligonucleotide probes (INFORM EBER Probe, Ventana) and the ISH iVIEW Blue Detection Kit (Ventana).

### 2.4. Statistics

Data for the 120 patients included in the study were transferred to a database. Necessary error checks and data cleaning were performed. The normal distribution of continuous variables (e.g., age and staining percentage) was evaluated using the Shapiro–Wilk test. All examined variables were found to be skewed and not normally distributed. Cross tables were created to examine the distribution of categorical variables according to CD47, and chi-squared values were calculated. Spearman’s rank correlation coefficient was calculated to investigate the relationships between variables. Given the nonnormal distribution of our data, nonparametric Mann–Whitney U tests were used. The statistical analysis and calculations were performed using IBM SPSS Statistics 22.0. A p-value <0.05 was considered indicative of a statistically significant difference.

## 3. Results

**Figure 1:**
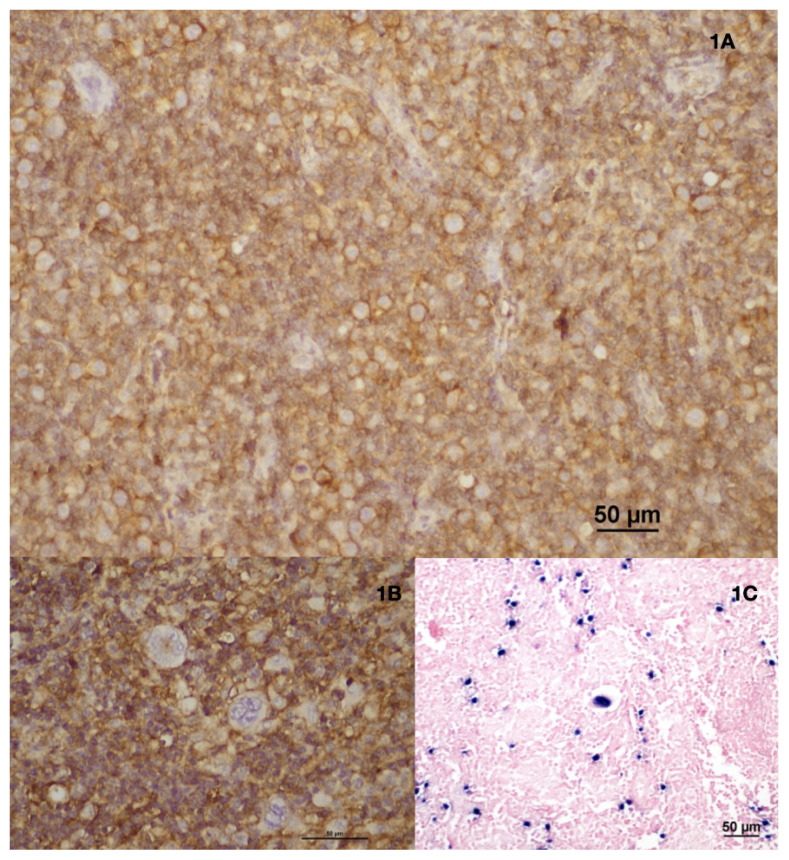
A. Strong membranous staining is observed in HRS cells using CD47 immunohistochemistry at 200× magnification. The staining highlights the robust expression of CD47 on the cell membrane, indicative of its significant presence on these malignant cells, B: A dot-like staining pattern is visible in the Golgi zone of a subset of HRS cells at 400× magnification, C: Nuclear positivity is widespread in HRS cells, as detected by Epstein–Barr virus-encoded RNA (EBER) in situ hybridization (ISH) at 200× magnification.

Of the patients, 76 (63.33%) were male and 44 (36.66%) were female, and their mean age was 38.77 years. CD47 staining showed cytoplasmic granular staining and dot-like staining in the Golgi zone in HRS cells (Figures 1A and 1B). EBV ISH test results were available for 93 patients; 48 (51.61%) were positive (Figure 1C) and 45 (48.39%) were negative.

The percentage of CD47 expression was determined by examining at least 20 HRS cells in the preparation. CD47 expression was observed in an average of 67% of HRS cells. Two groups were formed: one with a CD47 expression score ≤6 and the other with a score >6. Similarly, the patients were divided into two groups based on an IPI score of ≤2 or >2. A chi-squared test was used to examine the relationships between CD47, IPI, age, and sex.

**Figure 2:**
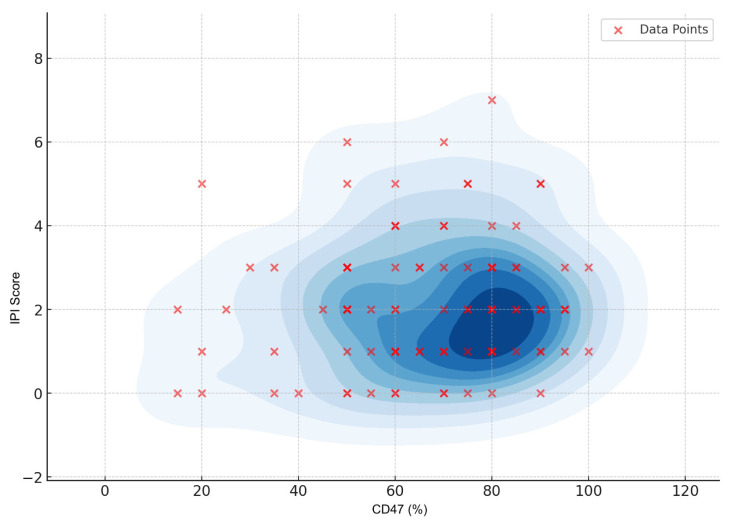
Comparison of CD47 staining score with IPI. The scatterplot shows the distribution of International Prognostic Index (IPI) scores as a function of CD47 expression (%). Each red cross represents an individual data point. The blue contour shading represents kernel density estimation (KDE), highlighting the concentration of data points within the sample population. This visualization demonstrates the overall distribution and density of the data points, suggesting a potential trend or relationship between CD47 expression and IPI score.

First, a chi-squared test between CD47 and IPI did not show a statistically significant association (χ^2^ = 0.061, p = 0.805, df = 1) (Figure 2). Similarly, no significant association was found between CD47 and EBV (χ^2^ = 0.790, p = 0.374, df = 1), age (χ^2^ = 0.00016, p = 0.990, df = 1), or CD47 and sex (χ^2^ = 2.95, p = 0.086, df = 1), although the latter approached the significance threshold (p values: 0.05) (Table).

Next, chi-squared tests were conducted to assess the relationships between IPI and other variables. No statistically significant associations were observed between IPI and age (χ^2^ = 0.00016, p = 0.990, df = 1) or IPI and sex (χ^2^ = 2.95, p = 0.086, df = 1), with all p-values exceeding the 0.05 significance threshold. Moreover, it was found that EBV positivity increased as the IPI score increased, and this was statistically significant (χ^2^ = 4.195, p = 0.041, df = 1). In summary, the statistical tests indicate that none of the clinical variables examined, including CD47, IPI, age, and sex, showed significant associations with one another in this cohort.

## 4. Discussion

The findings from the present study suggest that while CD47 is widely expressed in HRS cells in classical HL, its expression does not appear to correlate significantly with key clinical parameters such as age, sex, EBV status, or IPI score. This lack of significant association implies that CD47 expression alone may not be a reliable prognostic marker in classical HL. Previous studies have highlighted the role of CD47 as a “don’t eat me” signal, helping tumor cells evade phagocytosis by macrophages, which has led to its consideration as a potential therapeutic target [13,14]. However, our results indicate that despite the biological relevance of CD47 in immune evasion, its expression does not significantly influence clinical outcomes in HL, as measured by traditional prognostic markers like IPI.

The limitations of our study include a relatively small sample size, the lack of clinical follow-up data, and the absence of information on the response to classical treatments. These factors restrict the generalizability of our findings. Further research with larger cohorts and more comprehensive clinical data will be necessary to clarify the clinical significance of CD47.

CD47 was expressed in HRS cells predominantly in a dot-like pattern in the Golgi region, with most cases showing moderate to strong expression (80%), and an average expression rate of 67%. This distinct staining pattern allowed the differentiation of HRS cells from surrounding reactive cells. In a previous study by López-Pereira et al., CD30 and CD47 double immunofluorescence was identified as the best marker for HRS cells [15]. Other studies in the literature have also reported findings linking CD47 to tumor growth and metastasis in various types of lymphomas [16–18], suggesting that CD47 could potentially serve as a novel diagnostic marker for classical HL. A broader comparison of CD47’s clinical significance in other malignancies is warranted to contextualize its role in classical HL. In solid tumors such as ovarian cancer and non-small cell lung cancer, CD47 expression has been strongly associated with poorer prognosis and more aggressive disease due to its role in immune evasion [19,20]. Similarly, in hematologic malignancies like diffuse large B-cell lymphoma and acute myeloid leukemia, high CD47 expression has been correlated with worse clinical outcomes and resistance to treatment [21,22]. These findings underscore the variability in the prognostic relevance of CD47 across different malignancies and suggest that its role in classical HL may be more nuanced or context-specific. Further research exploring these interdisease differences could help clarify why CD47 shows prognostic significance in some cancers but not in classical HL.

Interestingly, while no significant relationship was found between CD47 and the clinical factors studied, a marginally significant trend was observed between higher IPI scores and increased EBV positivity (p = 0.041). This finding aligns with the existing literature, which associates EBV-positive HL with more aggressive disease and poorer prognosis [23,24]. No strong correlation was observed between CD47 expression and clinical parameters in our cohort, indicating that the prognostic significance of CD47 may be context-dependent and potentially more relevant in other malignancies or when analyzed in conjunction with additional biomarkers.

Given these findings, further research is necessary to fully elucidate the role of CD47 in HL. Future studies should consider larger patient cohorts and integrate additional biomarkers and clinical parameters to better understand the potential utility of CD47 as part of a composite prognostic model. Moreover, exploring the functional consequences of CD47 expression in HL, perhaps in the context of immune checkpoint inhibition or other immune-modulating therapies, may provide further insights into its role in the pathophysiology and treatment of this disease.

## 5. Conclusion

The present study demonstrated that CD47 was highly expressed in HRS cells in classical HL, but no significant correlation was found between CD47 expression and key clinical parameters such as age, sex, EBV status, or IPI score. Despite the established role of CD47 in immune evasion, its expression alone does not appear to significantly influence clinical outcomes in classical HL, at least as assessed by conventional prognostic markers.

While CD47 remains a promising therapeutic target in various malignancies, the results of our study suggest that its role in classical HL requires further clarification, particularly in larger cohorts with more comprehensive clinical data. Additionally, the significant association between EBV positivity and higher IPI scores aligns with the existing literature, suggesting that EBV may play a role in the progression of more aggressive forms of classical HL.

Future studies should focus on expanding sample sizes and incorporating long-term clinical outcomes to enhance the statistical power and generalizability of findings. Additionally, exploring the functional role of CD47 within the tumor microenvironment of classical HL is crucial. This includes investigating the efficacy of combining CD47-targeted therapies with immune checkpoint inhibitors, such as PD-1/PD-L1 blockers, to evaluate potential synergistic effects in enhancing antitumor immunity. Furthermore, examining the interactions between CD47 and various cellular elements, such as macrophages and T-cells, will elucidate their contributions to tumor immune evasion and disease progression.

## Figures and Tables

**Table:** The distribution of age, sex, IPI, EBER, and marker results according to CD47 coding for the 120 patients evaluated in the study.

	CD47		Total n (%)	Comparison Result
	≤6	>6		
	n(%)	n(%)		

Age				

≤65	40(52.6)	24(54.5)	64 (53.3)	x^2^ = 0.00016 p = 0.990

>65	36 (47.4)	20 (45.5)	56 (46.7)	

Sex				

Male	53(69.7)	23 (52.3)	76 (63.3)	x^2^ = 2.25 p = 0.086

Female	23 (30.3)	21(47.7)	44 (36.7)	

IPI				

≤2	48 (63.2)	26 (59.1)	74 (61.7)	x^2^ = 0.061 p = 0.805

>2	28 (36.8)	18 (40.9)	46 (38.3)	

EBV				

Positive	31 (55.4)	17 (45.9)	48 (51.6)	x^2^ = 0.790 p = 0.374

Negative	25 (44.6)	20 (54.1)	45 (48.4)	

## References

[1] Guru Murthy GS Szabo A Hamadani M Fenske TS Shah NN Contemporary outcomes for advanced-stage classical Hodgkin lymphoma in the U.S. analysis of surveillance, epidemiology, and results database Oncologist 2019 24 11 1488 1495 10.1634/theoncologist.2019-0027 31467178 PMC6853108

[2] Chen Y Zhu X Liu H Wang C Chen Y The application of HER2 and CD47 CAR-macrophage in ovarian cancer Journal of Translational Medicine 2023 21 1 654 10.1186/s12967-023-04572-4 37740183 PMC10517545

[3] Zhang S Liu X Li L Qiu L Qian Z Hodgkin’s lymphoma: 2023 update on treatment Cancer Biology Medicine 2023 21 4 269 273 10.20892/j.issn.2095-3941.2023.0378 38164723 PMC11033717

[4] Matozaki T Murata Y Okazawa H Ohnishi H Functions and molecular mechanisms of the CD47-SIRPα signaling pathway Trends in Cell Biology 2009 19 2 72 80 10.1016/j.tcb.2008.12.001 19144521

[5] Liu X Pu Y Cron K Deng L Kline J CD47 blockade triggers T cell-mediated destruction of immunogenic tumors Nature Medicine 2015 21 10 1209 1215 10.1038/nm.3931 PMC4598283 26322579

[6] Advani R Flinn I Popplewell L Forero A Bartlett NL CD47 blockade by Hu5F9-G4 and rituximab in non-Hodgkin’s lymphoma New England Journal of Medicine 2018 379 18 1711 1721 10.1056/NEJMoa1807315 30380386 PMC8058634

[7] Chao MP Alizadeh AA Tang C Myklebust JH Varghese B Anti-CD47 antibody synergizes with rituximab to promote phagocytosis and eradicate non-Hodgkin lymphoma *Cell* 2010 142 5 699 713 10.1016/j.cell.2010.07.044 20813259 PMC2943345

[8] Majeti R Chao MP Alizadeh AA Pang WW Jaiswal S CD47 is an adverse prognostic factor and therapeutic antibody target on human acute myeloid leukemia stem cells Cell 2009 138 2 286 299 10.1016/j.cell.2009.05.045 19632179 PMC2726837

[9] Lau APY Khavkine Binstock SS Thu KL CD47: The next frontier in immune checkpoint blockade for non-small cell lung cancer Cancers (Basel) 2023 15 21 654 10.3390/cancers15216554 37958404 PMC10649163

[10] Willingham SB Volkmer JP Gentles AJ Sahoo D Dalerba P The CD47-signal regulatory protein alpha (SIRPα) interaction is a therapeutic target for human solid tumors Proceedings of the National Academy of Sciences of the United States of America 2012 109 17 6662 6667 10.1073/pnas.1121623109 22451913 PMC3340046

[11] Depil S Morales O Auriault C Hodgkin’s disease and Epstein-Barr virus Annales de Biologie Clinique (Paris) 2004 62 6 639 648 10.1684/abc.2004.0719 15563422

[12] Murray PG Young LS An etiological role for the Epstein-Barr virus in the pathogenesis of classical Hodgkin lymphoma Blood 2019 134 7 591 596 10.1182/blood.2019000634 31186275

[13] Gholiha AR Hollander P Lof L Glimelius I Hedstrom G Checkpoint CD47 expression in classical Hodgkin lymphoma British Journal of Haematology 2022 197 5 580 589 10.1111/bjh.18272 35301709 PMC9310712

[14] Jia X Yan B Tian X Liu Q Jin CD47/SIRPα pathway mediates cancer immune escape and immunotherapy International Journal of Biological Sciences 2021 17 13 3281 3287 10.7150/ijbs.61381 34512146 PMC8416724

[15] Lopez-Pereira B Fernandez-Velasco AA Fernandez-Vega I Corte-Torres D Quiros C Expression of CD47 antigen in Reed-Sternberg cells as a new potential biomarker for classical Hodgkin lymphoma Clinical and Translational Oncology 2020 22 5 782 785 10.1007/s12094-019-02259-3 31359339

[16] Eladl E Tremblay-LeMay R Rastgoo N Musani R Chen W Role of CD47 in hematological malignancies Journal of Hematology and Oncology 2020 13 1 96 10.1186/s13045-020-00951-z 32677994 PMC7364564

[17] Kazama R Miyoshi H Takeuchi M Miyawaki K Nakashima K Combination of CD47 and signal-regulatory protein-alpha constituting the “don’t eat me signal” is a prognostic factor in diffuse large B-cell lymphoma Cancer Science 2020 111 7 2608 2619 10.1111/cas.14482 32342603 PMC7385345

[18] Subklewe M Bucklein V Sallman D Daver N Novel immunotherapies in the treatment of AML: is there hope? Hematology: American Society of Hematology Education Program 2023 2023 1 691 701 10.1182/hematology.2023000670 38066884 PMC10727092

[19] Willingham SB Volkmer JP Gentles AJ Sahoo D Dalerba The CD47-signal regulatory protein alpha (SIRPα) interaction is a therapeutic target for human solid tumors Proceedings of the National Academy of Sciences 2012 109 17 6662 6667 10.1073/pnas.1121623109 PMC3340046 22451913

[20] Zhang H Liu S Zhang J Wang Y CD47 expression in non-small cell lung cancer and its relationship with tumor-associated macrophage infiltration PLoS One 2024 19 12 e0314228 10.1371/journal.pone.0314228 39652550 PMC11627405

[21] Sammartano V Bestoso E Pacelli P Santoni A Sicuranza A Prognostic Impact of CD47 Overexpression in Patients with Acute Myeloid Leukemia Blood 2024 144 Supplement 1 6084 10.1182/blood-2024-194696

[22] Marra A Akarca AU Martino G Ramsay A Ascani S CD47 overexpression is common in intestinal non-GCB type diffuse large B-cell lymphoma Blood Advances 2022 6 24 6120 6130 10.1182/bloodadvances.2022008245 35475881 PMC9768246

[23] Hu J Zhang X Tao H Jia Y The prognostic value of Epstein-Barr virus infection in Hodgkin lymphoma: A systematic review and meta-analysis Frontiers in Oncology 2022 12 1034398 10.3389/fonc.2022.1034398 36387159 PMC9648611

[24] Wang C Zou SP Chen DG Wang JS Zheng YB Latent Epstein-Barr virus infection status and prognosis in patients with newly diagnosed Hodgkin lymphoma in Southeast China: A single-center retrospective study Hematology 2021 26 1 675 683 10.1080/16078454.2021.1938847 34493172

